# Supplementary material for: Low CD8 T Cell Counts Predict Benefit from Hypoxia-Modifying Therapy in Muscle-Invasive Bladder Cancer
Source: Cancers (Basel). 2022 Dec 21;15(1):41. doi: 10.3390/cancers15010041 (PMC9817934; doi:10.3390/cancers15010041)
Supplement: Supplementary file 1 [file cancers-15-00041-s001.zip › cancers-2059781-supplementary.pptx]

## Slide 1
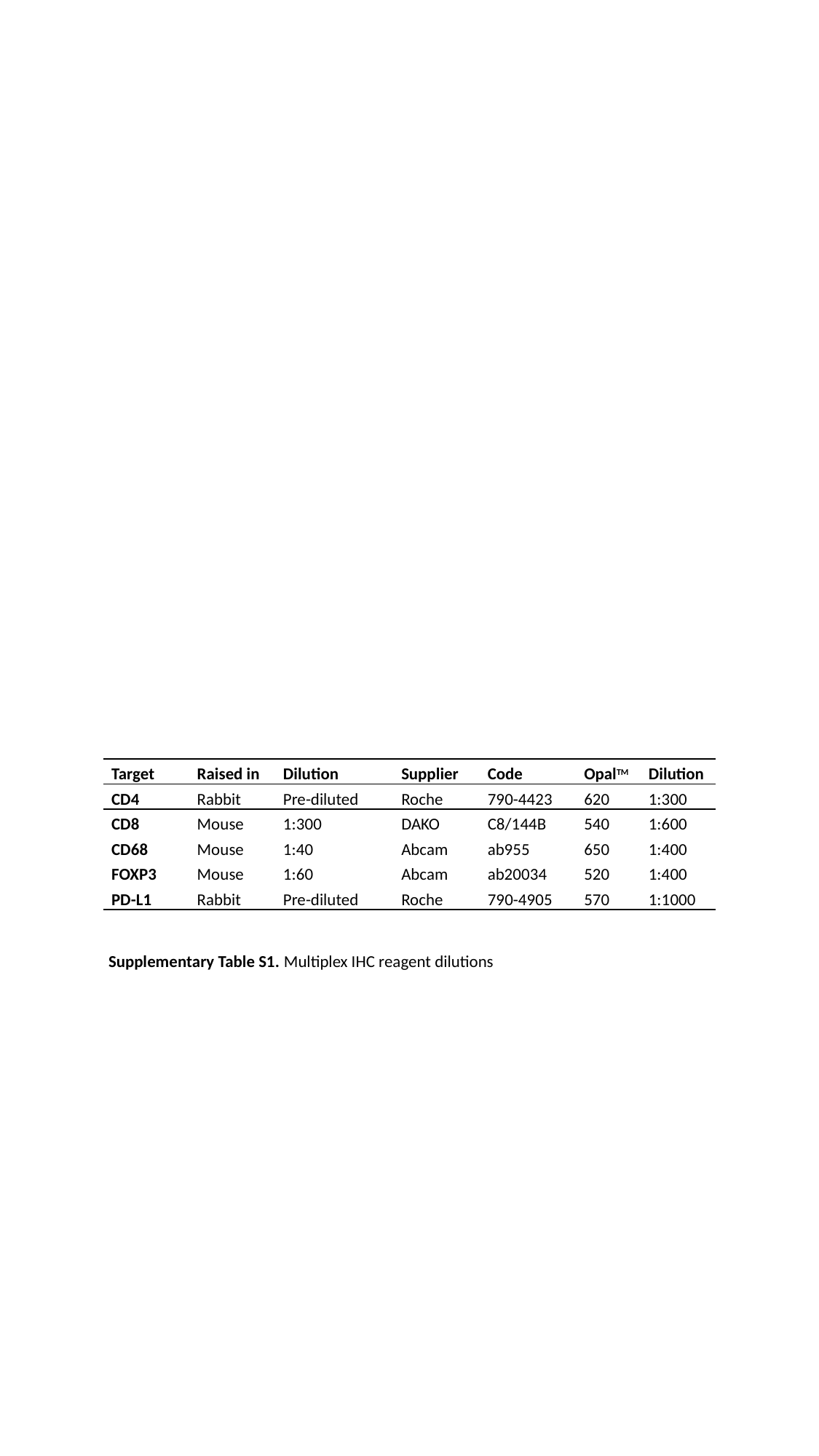

| Target | Raised in | Dilution | Supplier | Code | OpalTM | Dilution |
| --- | --- | --- | --- | --- | --- | --- |
| CD4 | Rabbit | Pre-diluted | Roche | 790-4423 | 620 | 1:300 |
| CD8 | Mouse | 1:300 | DAKO | C8/144B | 540 | 1:600 |
| CD68 | Mouse | 1:40 | Abcam | ab955 | 650 | 1:400 |
| FOXP3 | Mouse | 1:60 | Abcam | ab20034 | 520 | 1:400 |
| PD-L1 | Rabbit | Pre-diluted | Roche | 790-4905 | 570 | 1:1000 |
Supplementary Table S1. Multiplex IHC reagent dilutions

## Slide 2
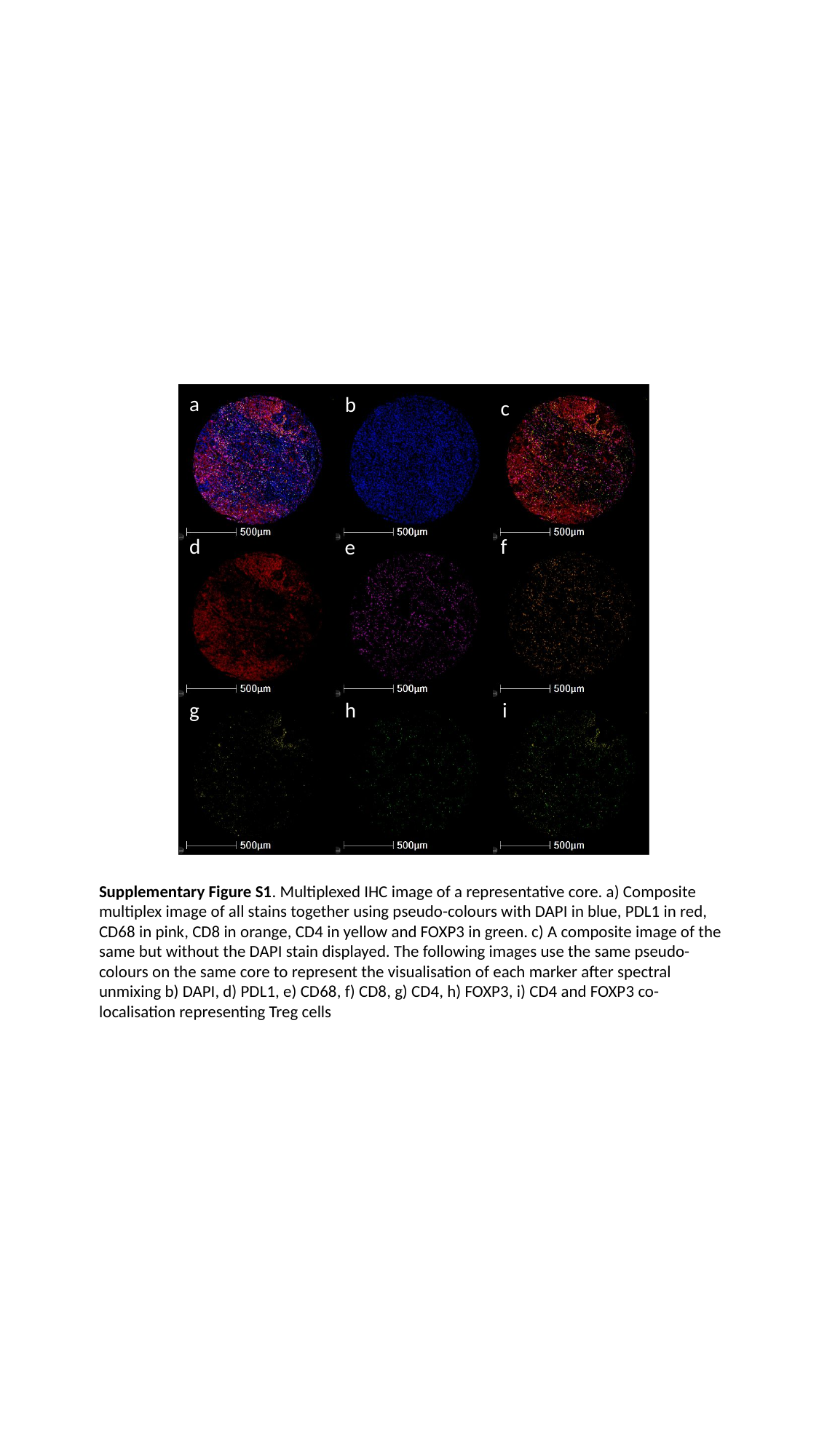

a
b
c
f
d
e
h
i
g
Supplementary Figure S1. Multiplexed IHC image of a representative core. a) Composite multiplex image of all stains together using pseudo-colours with DAPI in blue, PDL1 in red, CD68 in pink, CD8 in orange, CD4 in yellow and FOXP3 in green. c) A composite image of the same but without the DAPI stain displayed. The following images use the same pseudo-colours on the same core to represent the visualisation of each marker after spectral unmixing b) DAPI, d) PDL1, e) CD68, f) CD8, g) CD4, h) FOXP3, i) CD4 and FOXP3 co-localisation representing Treg cells

## Slide 3
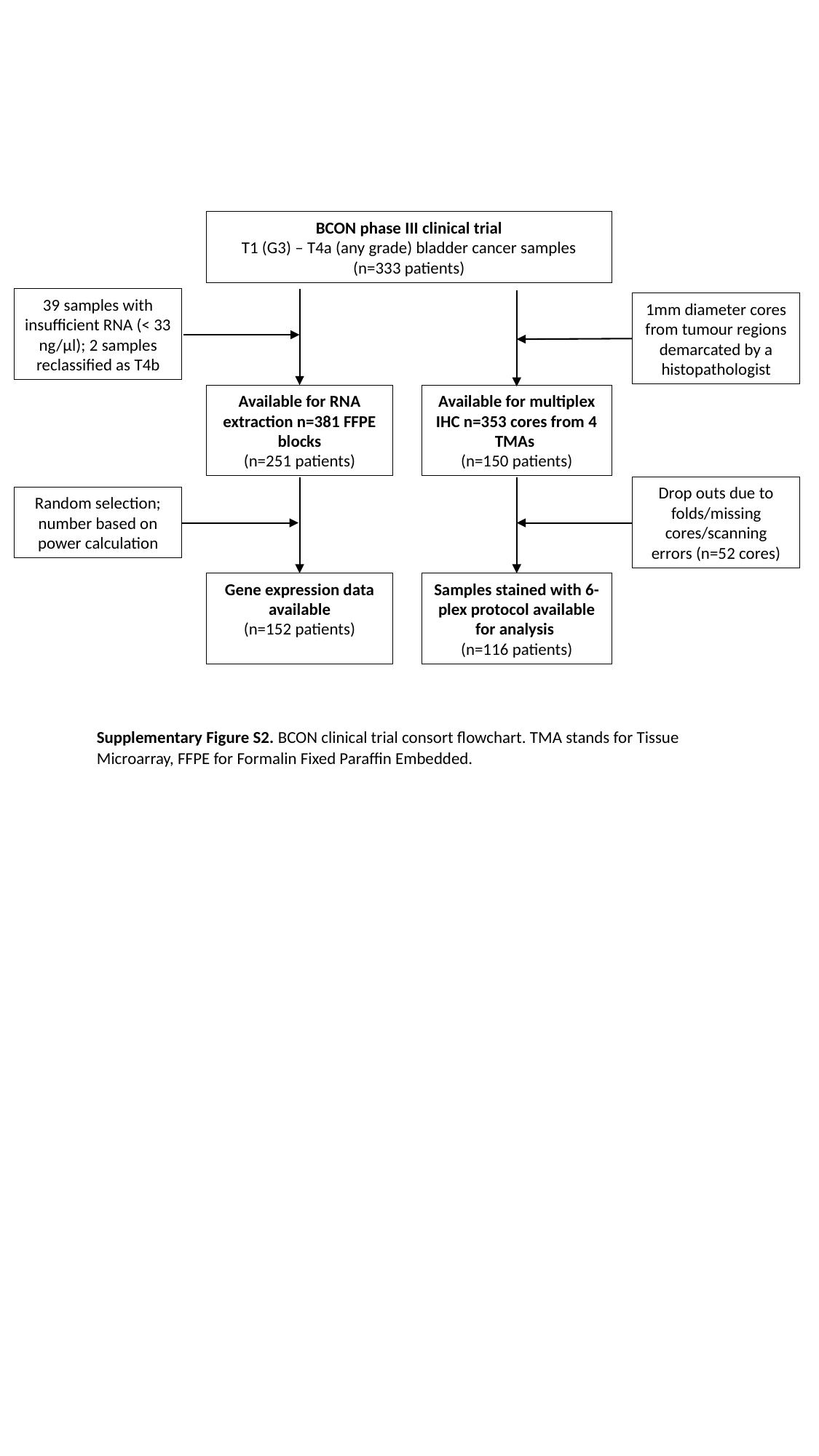

BCON phase III clinical trial
T1 (G3) – T4a (any grade) bladder cancer samples
(n=333 patients)
39 samples with insufficient RNA (< 33 ng/μl); 2 samples reclassified as T4b
1mm diameter cores from tumour regions demarcated by a histopathologist
Available for RNA extraction n=381 FFPE blocks
(n=251 patients)
Available for multiplex IHC n=353 cores from 4 TMAs
(n=150 patients)
Drop outs due to folds/missing cores/scanning errors (n=52 cores)
Random selection; number based on power calculation
Gene expression data available
(n=152 patients)
Samples stained with 6-plex protocol available for analysis
(n=116 patients)
Supplementary Figure S2. BCON clinical trial consort flowchart. TMA stands for Tissue Microarray, FFPE for Formalin Fixed Paraffin Embedded.

## Slide 4
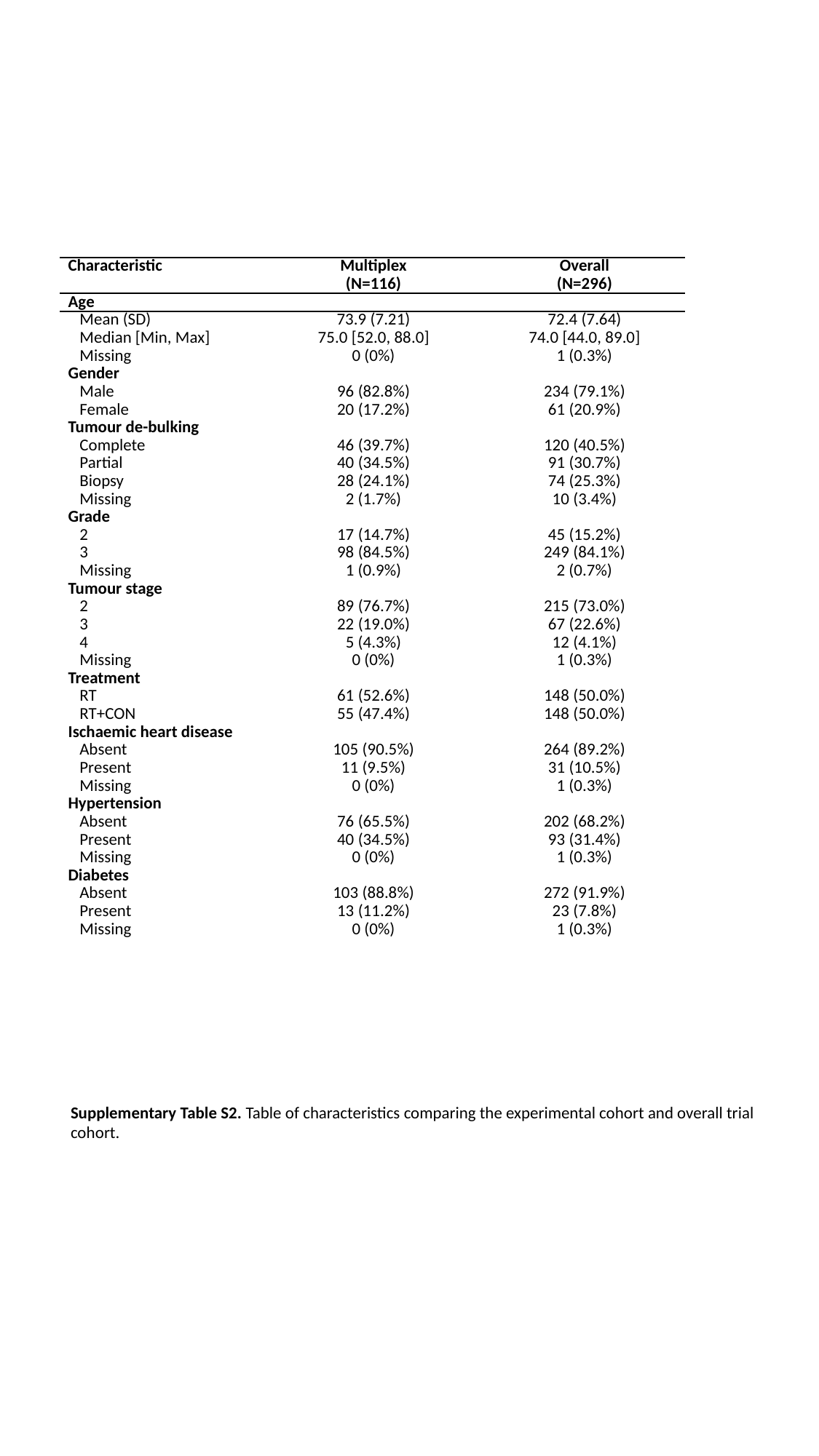

| Characteristic | Multiplex (N=116) | Overall (N=296) |
| --- | --- | --- |
| Age | | |
| Mean (SD) | 73.9 (7.21) | 72.4 (7.64) |
| Median [Min, Max] | 75.0 [52.0, 88.0] | 74.0 [44.0, 89.0] |
| Missing | 0 (0%) | 1 (0.3%) |
| Gender | | |
| Male | 96 (82.8%) | 234 (79.1%) |
| Female | 20 (17.2%) | 61 (20.9%) |
| Tumour de-bulking | | |
| Complete | 46 (39.7%) | 120 (40.5%) |
| Partial | 40 (34.5%) | 91 (30.7%) |
| Biopsy | 28 (24.1%) | 74 (25.3%) |
| Missing | 2 (1.7%) | 10 (3.4%) |
| Grade | | |
| 2 | 17 (14.7%) | 45 (15.2%) |
| 3 | 98 (84.5%) | 249 (84.1%) |
| Missing | 1 (0.9%) | 2 (0.7%) |
| Tumour stage | | |
| 2 | 89 (76.7%) | 215 (73.0%) |
| 3 | 22 (19.0%) | 67 (22.6%) |
| 4 | 5 (4.3%) | 12 (4.1%) |
| Missing | 0 (0%) | 1 (0.3%) |
| Treatment | | |
| RT | 61 (52.6%) | 148 (50.0%) |
| RT+CON | 55 (47.4%) | 148 (50.0%) |
| Ischaemic heart disease | | |
| Absent | 105 (90.5%) | 264 (89.2%) |
| Present | 11 (9.5%) | 31 (10.5%) |
| Missing | 0 (0%) | 1 (0.3%) |
| Hypertension | | |
| Absent | 76 (65.5%) | 202 (68.2%) |
| Present | 40 (34.5%) | 93 (31.4%) |
| Missing | 0 (0%) | 1 (0.3%) |
| Diabetes | | |
| Absent | 103 (88.8%) | 272 (91.9%) |
| Present | 13 (11.2%) | 23 (7.8%) |
| Missing | 0 (0%) | 1 (0.3%) |
Supplementary Table S2. Table of characteristics comparing the experimental cohort and overall trial cohort.

## Slide 5
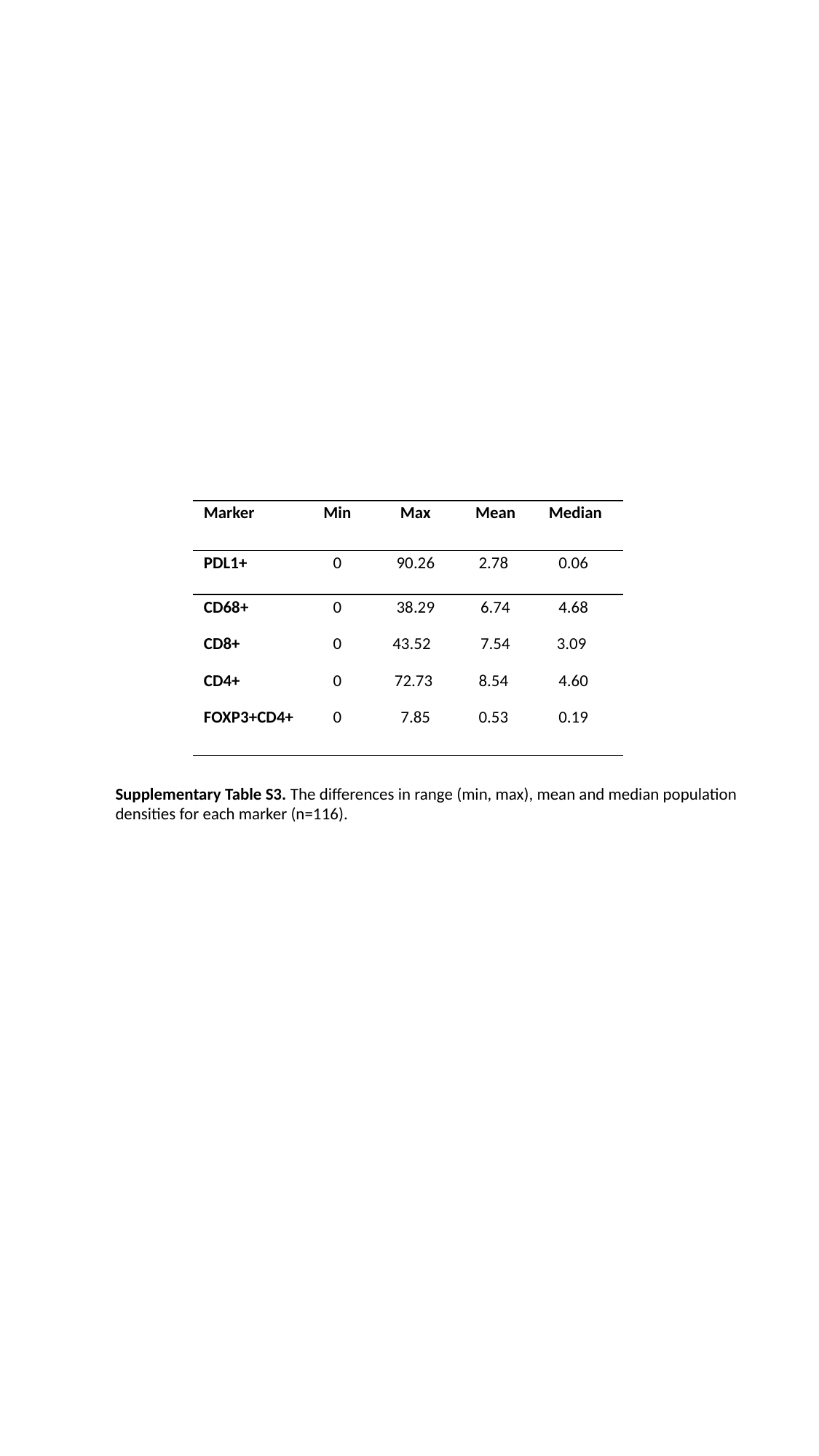

| Marker | Min | Max | Mean | Median |
| --- | --- | --- | --- | --- |
| PDL1+ | 0 | 90.26 | 2.78 | 0.06 |
| CD68+ | 0 | 38.29 | 6.74 | 4.68 |
| CD8+ | 0 | 43.52 | 7.54 | 3.09 |
| CD4+ | 0 | 72.73 | 8.54 | 4.60 |
| FOXP3+CD4+ | 0 | 7.85 | 0.53 | 0.19 |
Supplementary Table S3. The differences in range (min, max), mean and median population densities for each marker (n=116).

## Slide 6
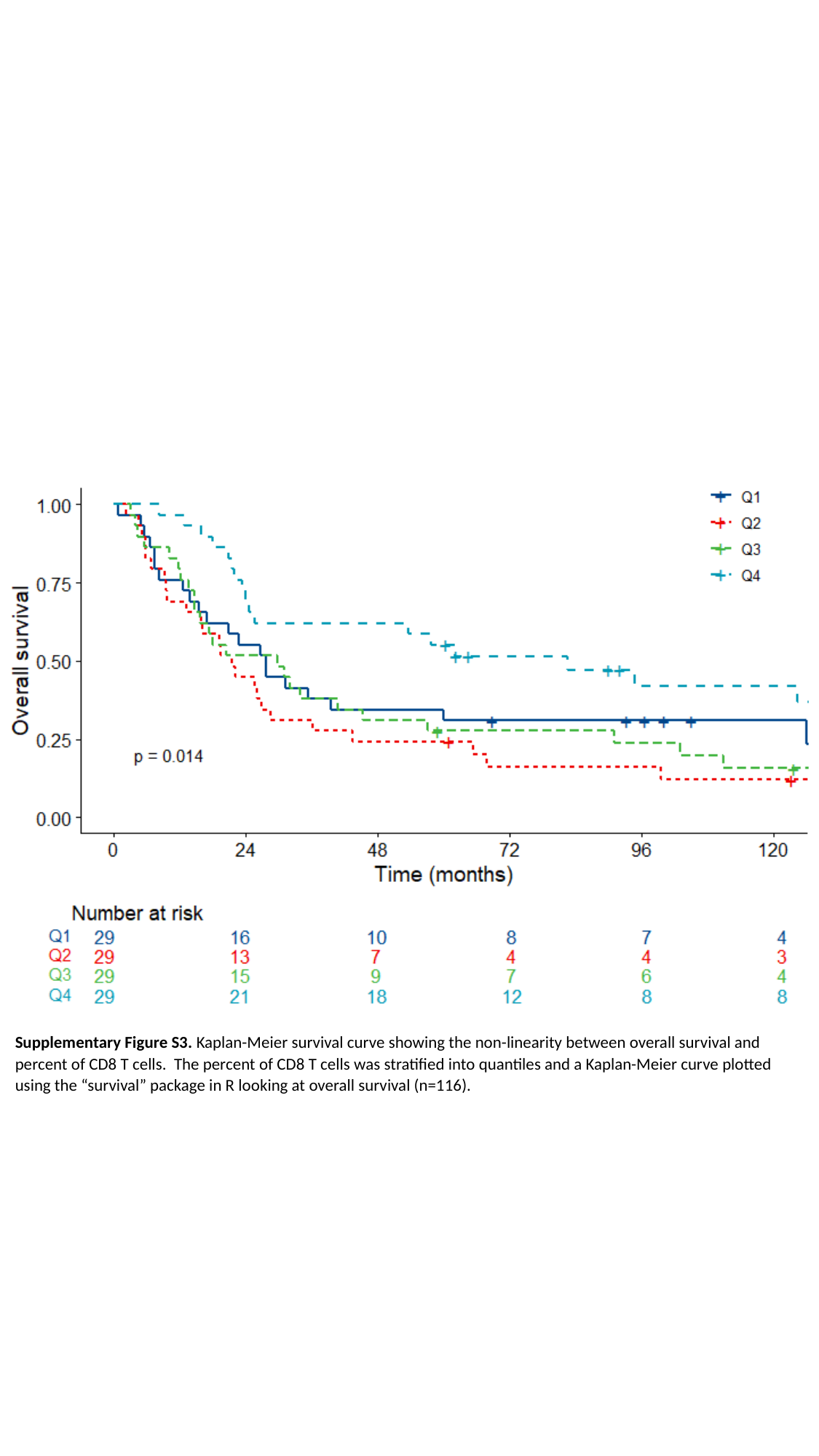

Supplementary Figure S3. Kaplan-Meier survival curve showing the non-linearity between overall survival and percent of CD8 T cells. The percent of CD8 T cells was stratified into quantiles and a Kaplan-Meier curve plotted using the “survival” package in R looking at overall survival (n=116).

## Slide 7
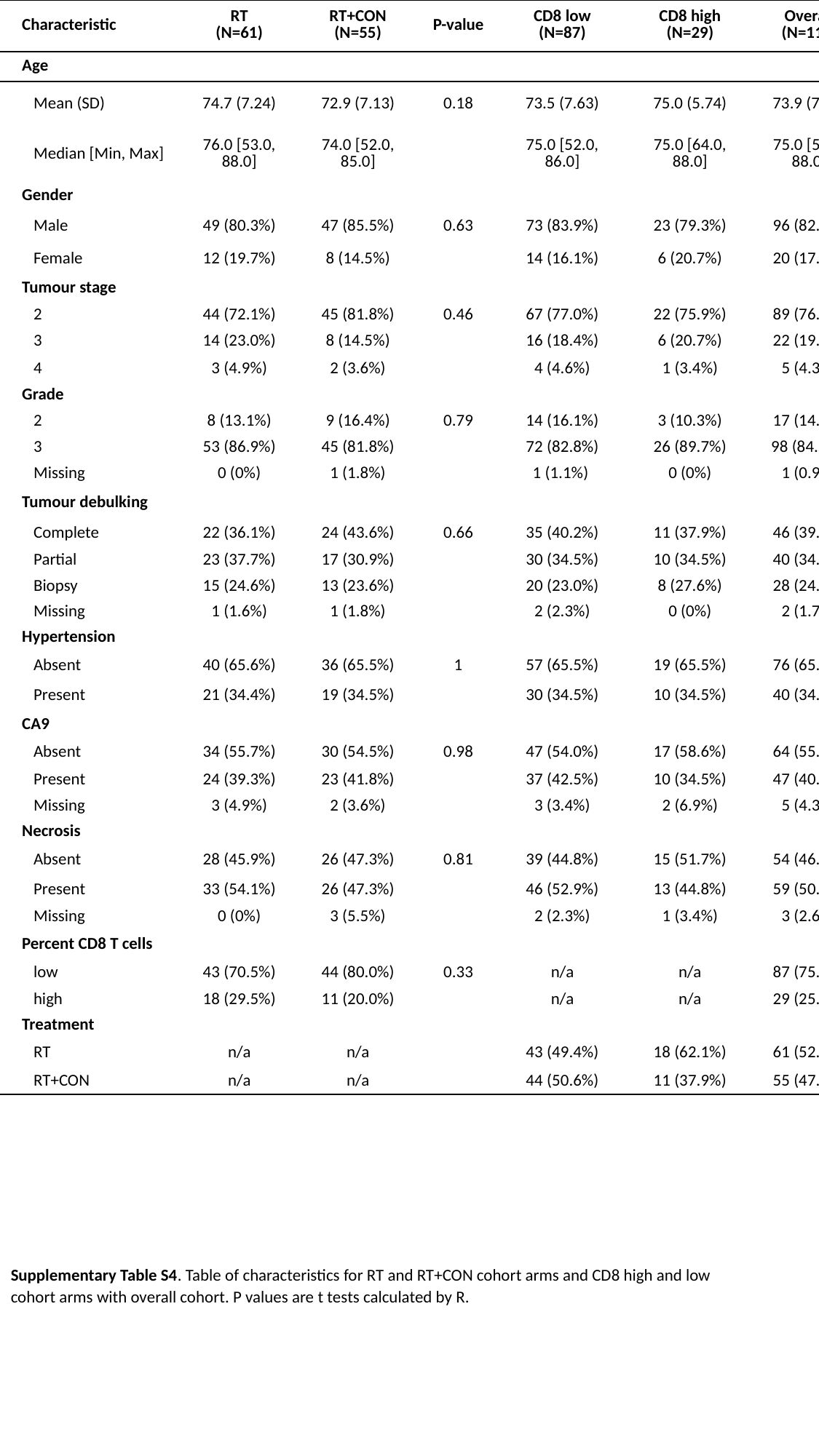

| Characteristic | RT (N=61) | RT+CON (N=55) | P-value | CD8 low (N=87) | CD8 high (N=29) | Overall (N=116) | P-value |
| --- | --- | --- | --- | --- | --- | --- | --- |
| Age | | | | | | | |
| Mean (SD) | 74.7 (7.24) | 72.9 (7.13) | 0.18 | 73.5 (7.63) | 75.0 (5.74) | 73.9 (7.21) | 0.27 |
| Median [Min, Max] | 76.0 [53.0, 88.0] | 74.0 [52.0, 85.0] | | 75.0 [52.0, 86.0] | 75.0 [64.0, 88.0] | 75.0 [52.0, 88.0] | |
| Gender | | | | | | | |
| Male | 49 (80.3%) | 47 (85.5%) | 0.63 | 73 (83.9%) | 23 (79.3%) | 96 (82.8%) | 0.78 |
| Female | 12 (19.7%) | 8 (14.5%) | | 14 (16.1%) | 6 (20.7%) | 20 (17.2%) | |
| Tumour stage | | | | | | | |
| 2 | 44 (72.1%) | 45 (81.8%) | 0.46 | 67 (77.0%) | 22 (75.9%) | 89 (76.7%) | 0.94 |
| 3 | 14 (23.0%) | 8 (14.5%) | | 16 (18.4%) | 6 (20.7%) | 22 (19.0%) | |
| 4 | 3 (4.9%) | 2 (3.6%) | | 4 (4.6%) | 1 (3.4%) | 5 (4.3%) | |
| Grade | | | | | | | |
| 2 | 8 (13.1%) | 9 (16.4%) | 0.79 | 14 (16.1%) | 3 (10.3%) | 17 (14.7%) | 0.63 |
| 3 | 53 (86.9%) | 45 (81.8%) | | 72 (82.8%) | 26 (89.7%) | 98 (84.5%) | |
| Missing | 0 (0%) | 1 (1.8%) | | 1 (1.1%) | 0 (0%) | 1 (0.9%) | |
| Tumour debulking | | | | | | | |
| Complete | 22 (36.1%) | 24 (43.6%) | 0.66 | 35 (40.2%) | 11 (37.9%) | 46 (39.7%) | 0.9 |
| Partial | 23 (37.7%) | 17 (30.9%) | | 30 (34.5%) | 10 (34.5%) | 40 (34.5%) | |
| Biopsy | 15 (24.6%) | 13 (23.6%) | | 20 (23.0%) | 8 (27.6%) | 28 (24.1%) | |
| Missing | 1 (1.6%) | 1 (1.8%) | | 2 (2.3%) | 0 (0%) | 2 (1.7%) | |
| Hypertension | | | | | | | |
| Absent | 40 (65.6%) | 36 (65.5%) | 1 | 57 (65.5%) | 19 (65.5%) | 76 (65.5%) | 1 |
| Present | 21 (34.4%) | 19 (34.5%) | | 30 (34.5%) | 10 (34.5%) | 40 (34.5%) | |
| CA9 | | | | | | | |
| Absent | 34 (55.7%) | 30 (54.5%) | 0.98 | 47 (54.0%) | 17 (58.6%) | 64 (55.2%) | 0.68 |
| Present | 24 (39.3%) | 23 (41.8%) | | 37 (42.5%) | 10 (34.5%) | 47 (40.5%) | |
| Missing | 3 (4.9%) | 2 (3.6%) | | 3 (3.4%) | 2 (6.9%) | 5 (4.3%) | |
| Necrosis | | | | | | | |
| Absent | 28 (45.9%) | 26 (47.3%) | 0.81 | 39 (44.8%) | 15 (51.7%) | 54 (46.6%) | 0.63 |
| Present | 33 (54.1%) | 26 (47.3%) | | 46 (52.9%) | 13 (44.8%) | 59 (50.9%) | |
| Missing | 0 (0%) | 3 (5.5%) | | 2 (2.3%) | 1 (3.4%) | 3 (2.6%) | |
| Percent CD8 T cells | | | | | | | |
| low | 43 (70.5%) | 44 (80.0%) | 0.33 | n/a | n/a | 87 (75.0%) | |
| high | 18 (29.5%) | 11 (20.0%) | | n/a | n/a | 29 (25.0%) | |
| Treatment | | | | | | | |
| RT | n/a | n/a | | 43 (49.4%) | 18 (62.1%) | 61 (52.6%) | 0.33 |
| RT+CON | n/a | n/a | | 44 (50.6%) | 11 (37.9%) | 55 (47.4%) | |
Supplementary Table S4. Table of characteristics for RT and RT+CON cohort arms and CD8 high and low cohort arms with overall cohort. P values are t tests calculated by R.

## Slide 8
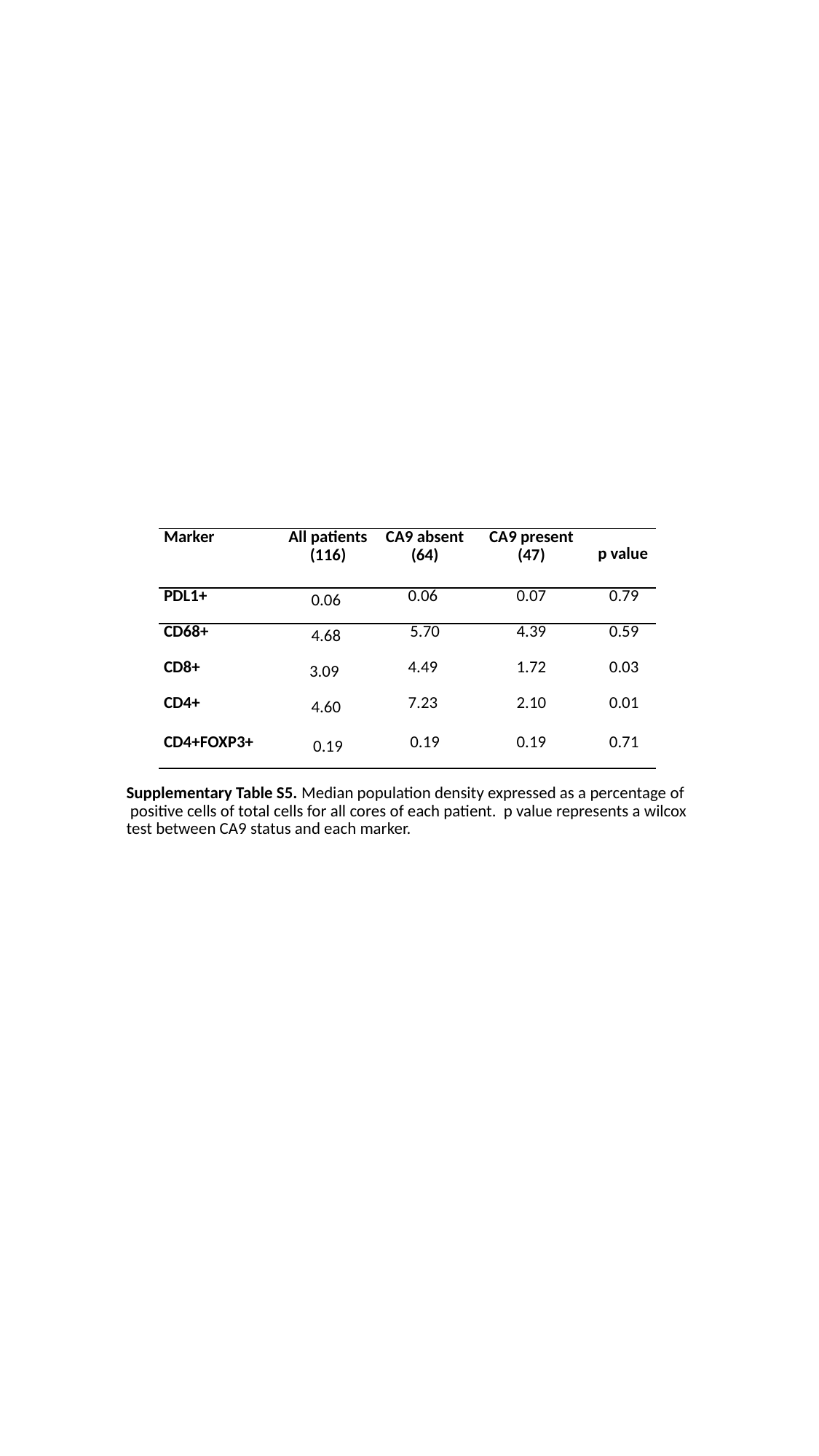

| Marker | All patients (116) | CA9 absent (64) | CA9 present (47) | p value |
| --- | --- | --- | --- | --- |
| PDL1+ | 0.06 | 0.06 | 0.07 | 0.79 |
| CD68+ | 4.68 | 5.70 | 4.39 | 0.59 |
| CD8+ | 3.09 | 4.49 | 1.72 | 0.03 |
| CD4+ | 4.60 | 7.23 | 2.10 | 0.01 |
| CD4+FOXP3+ | 0.19 | 0.19 | 0.19 | 0.71 |
| Supplementary Table S5. Median population density expressed as a percentage of positive cells of total cells for all cores of each patient. p value represents a wilcox test between CA9 status and each marker. |
| --- |

## Slide 9
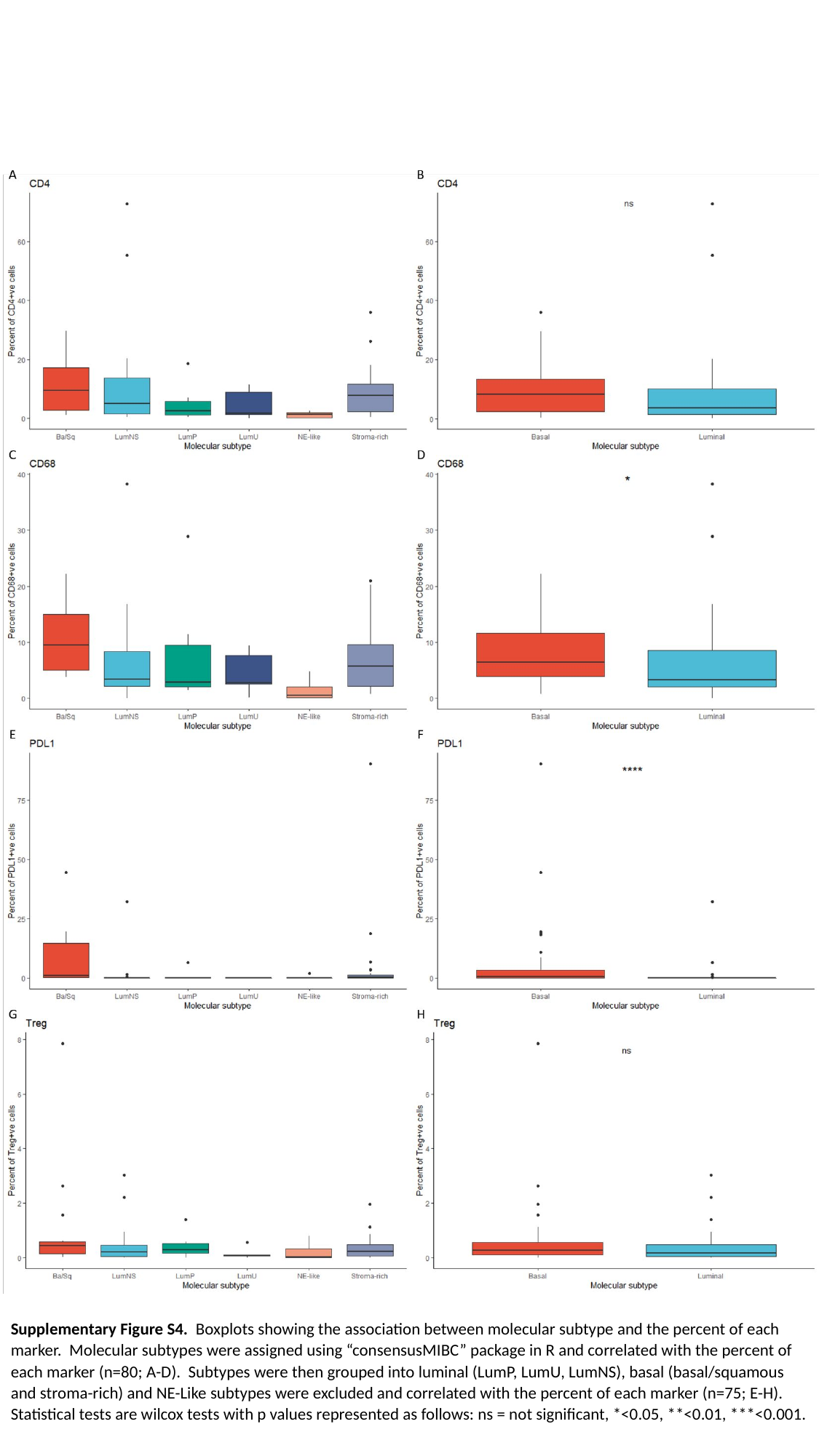

Supplementary Figure S4. Boxplots showing the association between molecular subtype and the percent of each marker. Molecular subtypes were assigned using “consensusMIBC” package in R and correlated with the percent of each marker (n=80; A-D). Subtypes were then grouped into luminal (LumP, LumU, LumNS), basal (basal/squamous and stroma-rich) and NE-Like subtypes were excluded and correlated with the percent of each marker (n=75; E-H). Statistical tests are wilcox tests with p values represented as follows: ns = not significant, *<0.05, **<0.01, ***<0.001.
